# The immune response of bats differs between pre-migration and migration seasons

**DOI:** 10.1038/s41598-020-74473-3

**Published:** 2020-10-15

**Authors:** Christian C. Voigt, Marcus Fritze, Oliver Lindecke, David Costantini, Gunārs Pētersons, Gábor Á. Czirják

**Affiliations:** 1 Leibniz Institute for Zoo and Wildlife Research, Alfred-Kowalke-Str. 17, 10315 Berlin, Germany; 2 Institute of Biology, Freie Universität Berlin, Takustr. 6, 14895 Berlin, Germany; 3 Unité Physiologie Moléculaire et Adaptation (PhyMA), Muséum National d’Histoire Naturelle, CNRS, 57 Rue Cuvier, CP32, 75005 Paris, France; 4 Faculty of Veterinary Medicine, Latvia University of Life Sciences and Technologies, K. Helmana street 8, Jelgava, 3004 Latvia

**Keywords:** Ecology, Immunology, Zoology, Ecology

## Abstract

Maintaining a competent immune system is energetically costly and thus immunity may be traded against other costly traits such as seasonal migration. Here, we tested in long-distance migratory Nathusius’ pipistrelles (*Pipistrellus nathusii*), if selected branches of immunity are expressed differently in response to the energy demands and oxidative stress of aerial migration. During the migration period, we observed higher baseline lymphocyte and lower neutrophil levels than during the pre-migration period, but no stronger response of cellular effectors to an antigen challenge. Baseline plasma haptoglobin, as a component of the humoral innate immunity, remained similar during both seasons, yet baseline plasma haptoglobin levels increased by a factor of 7.8 in migratory bats during an immune challenge, whereas they did not change during the pre-migration period. Oxidative stress was higher during migration than during pre-migration, yet there was no association between blood oxidative status and immune parameters, and immune challenge did not trigger any changes in oxidative stress, irrespective of season. Our findings suggest that humoral effectors of the acute phase response may play a stronger role in the first-line defense against infections for migrating bats compared to non-migrating bats. We conclude that Nathusius’ pipistrelles allocate resources differently into the branches of their immune system, most likely following current demands resulting from tight energy budgets during migration.

## Introduction

All animals use their immune system to protect themselves against infections. This defence barrier against pathogens involves metabolic costs for its production, maintenance and usage^1–4^. For example, resting metabolic rates increase when vertebrates activate their immune system following an experimental infection or antigen challenge^4–6^. Immunity may also incite other costs, such as increased molecular oxidative damage due to the release of reactive oxygen species by immune cells^7^. Thus, the functioning of the immune system might get traded against other metabolically costly traits or physiological functions such as reproduction, movement or thermoregulation when energy is limited^8^.

Migration of terrestrial vertebrates is energetically costly and also causes high levels of oxidative stress^9–12^. Therefore, seasonal journeys may be crucial trade-off situations during which a migrant is forced to balance the need for a competent immunity and the necessity to travel long distances to escape adverse ambient conditions at the place of origin or to reach favourable habitats for establishing territories prior to conspecifics^13–15^. Previous studies suggested that migratory animals may suppress immunity in favour of the energy demands of locomotion^16–20^. In addition, Owen and Moore observed that alleviated immunity in birds arriving at a stopover site coincided with poor body condition^21^. Oxidative stress generated by intense exercise might even further constrain an animal’s immunity by damaging the immune cells or by triggering immune-regulatory effects. For example, pre-challenge levels of the free radical superoxide were negatively related to the strength of the subsequent immune response towards lipopolysaccharide (LPS) in painted dragon lizards *Ctenophorus pictus*^22^. Similarly, long-lived mouse mutants with reduced mitochondrial superoxide production showed an enhanced inflammatory response^23^, whereas those with elevated mitochondrial superoxide experienced both an impaired T-cell development and function^24^. Further, production of antioxidants seems to be traded against the constitutive innate immunity, measured by bacterial killing capacity, in migratory blackbirds^19^. In general, a suppressed immunity in migratory animals may come at high risk, since migrants may be exposed to a larger variety of pathogens. Accordingly, it has also been suggested that migratory animals may have to boost their immunity to prepare for novel or a large variety of pathogens along their journeys^25^.

Although not widely recognized as migratory, some species of bats may cover hundreds or even thousands of kilometres during their seasonal journeys from their summer breeding areas to wintering sites^26–29^. Bats are efficient migrants owing to their ability of flapping flight; a unique feature among mammals. Bat flight is energetically costly, exceeding usually about ten times basal metabolic rate^12,30,31^. Therefore, it is likely that bats encounter high energy demands along their migratory journeys, which suggests that they have to balance a tight energy budget between various needs. Indeed, migratory bats optimize flight energy expenditure by travelling at a flight speed at which they spend the least amount of energy for the distance covered^12^. Besides selecting an optimal flight speed, bats may also save energy by entering daily torpor at stopover sites^32^.

Currently, it is unknown if bats trade body functions, such as their immunity, against the high metabolic costs of locomotion during migration. Bats may, for example, experience increased oxidative damage while migrating^11^, which might constrain the immune function even further by direct effects, such as damaging immune cells, and indirect effects, such as having to allocate resources away from immunity and into the production of costly antioxidants. Indeed, bats seem to have a high capacity to mitigate oxidative damages, which might partly explain why bats are unusually long lived mammals^33–35^ and repair DNA oxidation^36^. Frugivorous bats appear to be better equipped to resist oxidative stress than insectivorous bats^37^. Nonetheless, immune challenges^38^, migratory flights^11^, and environmental factors^39^ may influence oxidative stress in bats. Here, we asked if branches of a bat’s immunity are suppressed during migration compared to the pre-migration period. We measured baseline levels of both cellular and humoral effectors of the innate immunity (relative neutrophil numbers and haptoglobin concentration, respectively), the cellular effector of adaptive immunity (lymphocyte numbers) and on how these effectors responded to an antigen challenge. We also asked if increased plasma oxidative stress is associated with reduced immune markers, indicating a physiological link between oxidative stress and immunity. Migratory bats are particularly rewarding as a model to study putative immune suppression, because the order of Chiroptera is known to host relatively large numbers of pathogens^40–42^, some of which may have zoonotic potential^43,44^. Thus, an impaired immunity could be relevant for the spread of pathogens in this highly mobile taxon.

Our study species, Nathusius’ pipistrelles (*Pipistrellus nathusii*), is a bat with a record of long-distance movements across continental Europe^45^. Nathusius’ pipistrelles may migrate from Northeastern Europe, e.g. Baltic countries, Fennoscandia, Belarus and Russia, to Central and Southwestern Europe^45^. In general, the annual cycle of *P. nathusii* can be separated into five distinct phases: the hibernation period (October to early April), spring migration (late April, May), pregnancy and lactation (May to June), the pre-migration period (July) and summer migration (August–September)^45^. In our study, we focused on two periods: the pre-migration and summer migration period. During the pre-migration period, females form larger colonies of 5–15 individuals where they reproduced before. Usually, juveniles are weaned by mid-July yet remain with the female group until migration. Before migration, male *P. nathusii* remain mostly isolated in their roosts and do not join larger colonies. During the migration period, male and female Nathusius’ pipistrelles are encountered at about the same rate at our study site at the Latvian coastline^45^.

We tested two hypotheses related to the immunity and one related to the potentially constraining effect of oxidative stress on the immunity of migratory bats. Based on the putative energetic costs of the various immune branches^14,15,46^, we first predicted for *P. nathusii* that the baseline levels of the innate immunity (both cellular and humoral aspects) are elevated during the migration period compared to the pre-migration period, since relying on non-specific, quick immunity is also beneficial when encountering novel and more diverse pathogens during migratory journeys. Second, we studied the acute phase response of bats to an antigen challenge by comparing cellular and humoral parameters between animals challenged with LPS and control animals across seasons. In line with our previous hypothesis, we predicted that migratory bats would respond stronger to the immune challenge compared with the pre-migrating bats and that the response would be mainly driven by humoral effectors.

Finally, if oxidative stress works as an additional constraint for the immunity of migratory bats, we predicted that oxidative stress would be higher in migratory bats and negatively correlated with immune markers. Considering the elevated levels of oxidative stress in Nathusius’ pipistrelles during migratory flights^11^, we expected a weaker effect of a simulated bacterial infection on markers of the oxidative status in migratory Nathusius’ pipistrelles than in pre-migratory conspecifics to avoid overly high physiological costs while migrating.

## Results

### Baseline immunological parameters

**Figure 1:**
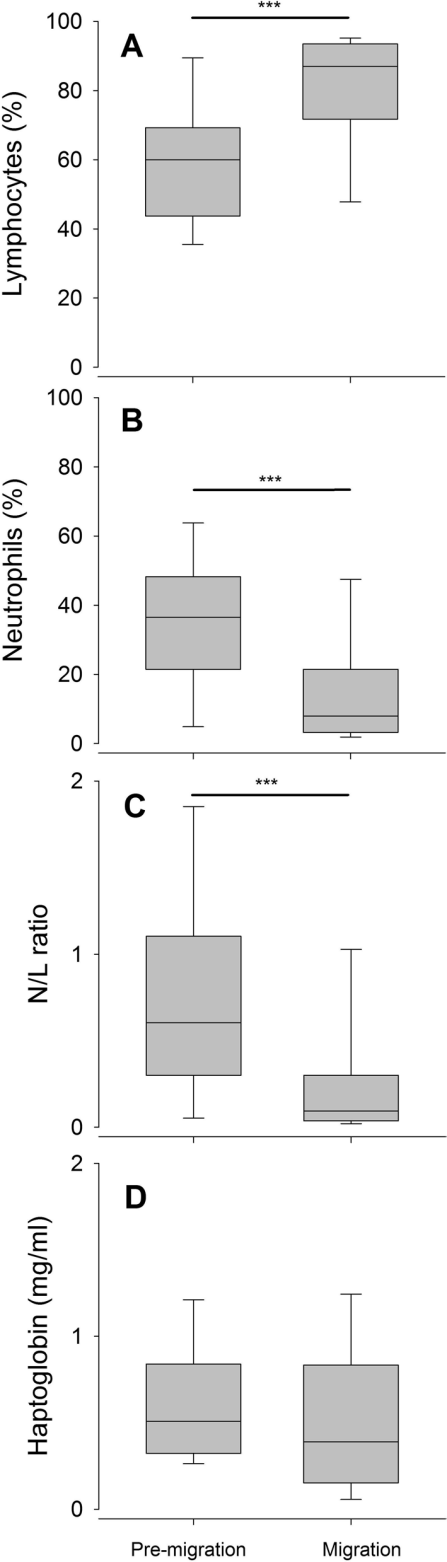
Differences in the proportions of white blood cells (%; **A**, lymphocytes = L; **B**, neutrophils = N), N/L ratios **(C)** and haptoglobin concentration (mg ml^−1^, **D**) between pre-migration and migration season in *P. nathusii*. Asterisks above horizontal lines between groups (pre-migration and migration) indicate significant seasonal changes (* = p < 0.05, ** = p < 0.005, *** = p < 0.001).

**Figure 2:**
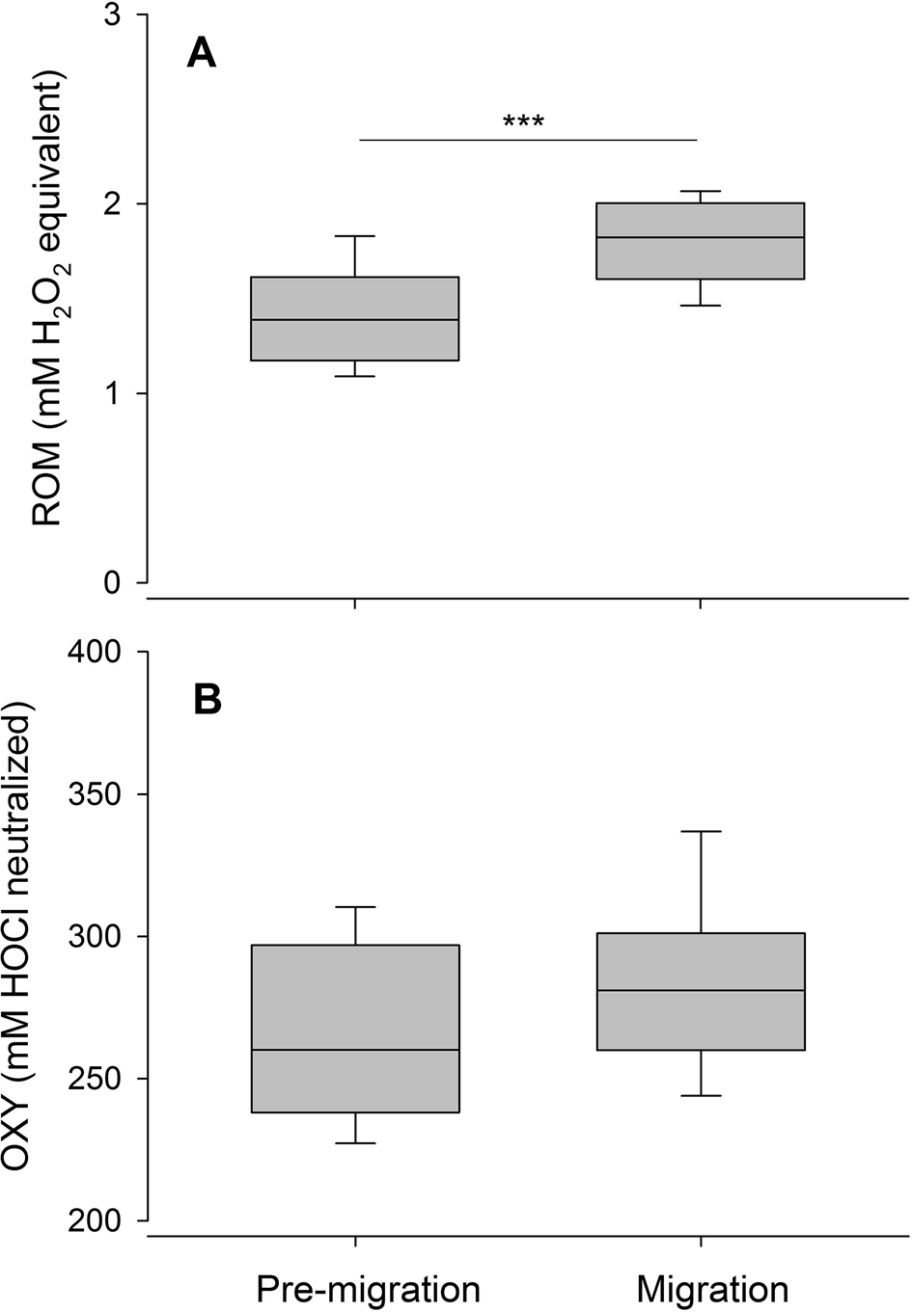
Differences in primary plasma oxidative damage products measured as ROMs (mM H_2_0_2_ equivalents; **(A)** and plasma non-enzymatic antioxidant capacity measured as OXY (mM HOCl neutralized, **(B)** between pre-migration and migration season in *P. nathusii*. Asterisks above horizontal lines between groups (pre-migration and migration) indicate significant seasonal changes (* = p < 0.05, ** = p < 0.005, *** = p < 0.001).

Body masses of *Pipistrellus nathusii* did not differ between pre-migration (7.8 ± 0.6 g; mean ± standard deviation) and migration period (7.4 ± 0.9 g; Wilcoxon Mann–Whitney U test: W = 216, p = 0.42) prior experimental manipulation. For assessing the cellular immunity of bats, we conducted differential white blood cell counts (see supplementary material for raw data). The proportion of lymphocytes (L) were higher in Nathusius’ pipistrelles during the migration than during the pre-migration season (Table 1, Fig. 1A), whereas the proportion of neutrophils (N) was significantly lower during the migration than during the pre-migration season (Table 1, Fig. 1B). As a result, N/L ratios were significantly higher in Nathusius’ pipistrelles during the pre-migration period compared to conspecifics during the migration period (Table 1, Fig. 1C). As a measure of the innate humoral immunity, we analysed haptoglobin concentrations in blood plasma using colorimetric methods. We could not find a significant difference between seasons (Table 1, Fig. 1C). The oxidative status of plasma was assessed by measuring one marker of non-enzymatic antioxidant capacity (OXY) and one marker of plasma oxidative damage (reactive oxygen metabolites; ROM) in blood plasma using conventional assays (supplementary material). We found a higher oxidative damage (i.e., ROMs) in *P. nathusii* during the migration compared to conspecifics in the pre-migration period (Table 1, Fig. 2A). However, plasma OXY concentrations were similar in Nathusius’ pipistrelles across seasons (Table 1, Fig. 2B). ROMs were not correlated with baseline lymphocyte percentages of both pre-migration (rho = − 0.11, p = 0.659) and migration season (rho = 0.35, p = 0.110). We also did not find correlations between ROMs and neutrophil percentages (pre-migration: rho = 0.12, p = 0.632; migration: rho = − 0.27, p = 0.218) as well as between ROMs and plasma haptoglobin concentrations (pre-migration: rho = 0.19, p = 0.44; migration: rho = 0.31, p = 0.179).

**Table 1:** Baseline immunological parameters and measure of oxidative status in *P. nathusii* captured in Latvia during the pre-migration (n = 18 females) and migration period (11 females, 17 males). Values indicate mean ± one standard deviation (median).

	Pre-migration	Migration	Test statistics	P-value
Lymphocytes (%)	58.7 ± 19.2 (60.0)	79.9 ± 17.2 (87)	402.5	< 0.001
Neutrophils (%)	36.0 ± 19.2 (36.5)	15.6 ± 16.8 (8.0)	101	< 0.001
N/L ratio	0.27 ± 0.37 (0.09)	0.82 ± 0.77 (0.60)	102.5	< 0.001
Haptoglobin (mg/ml)	0.61 ± 0.36 (0.51)	0.49 ± 0.41 (0.39)	186	0.59
ROMs (mM H_2_0_2_ equivalents	1.61 ± 0.47 (1.51)	1.87 ± 0.13 (1.85)	339	< 0.001
OXY (mM HOCl neutralized)	269.2 ± 36.5 (260.8)	277.7 ± 28.4 (272.9)	308	0.079

### Acute phase response to an immune challenge

**Figure 3:**
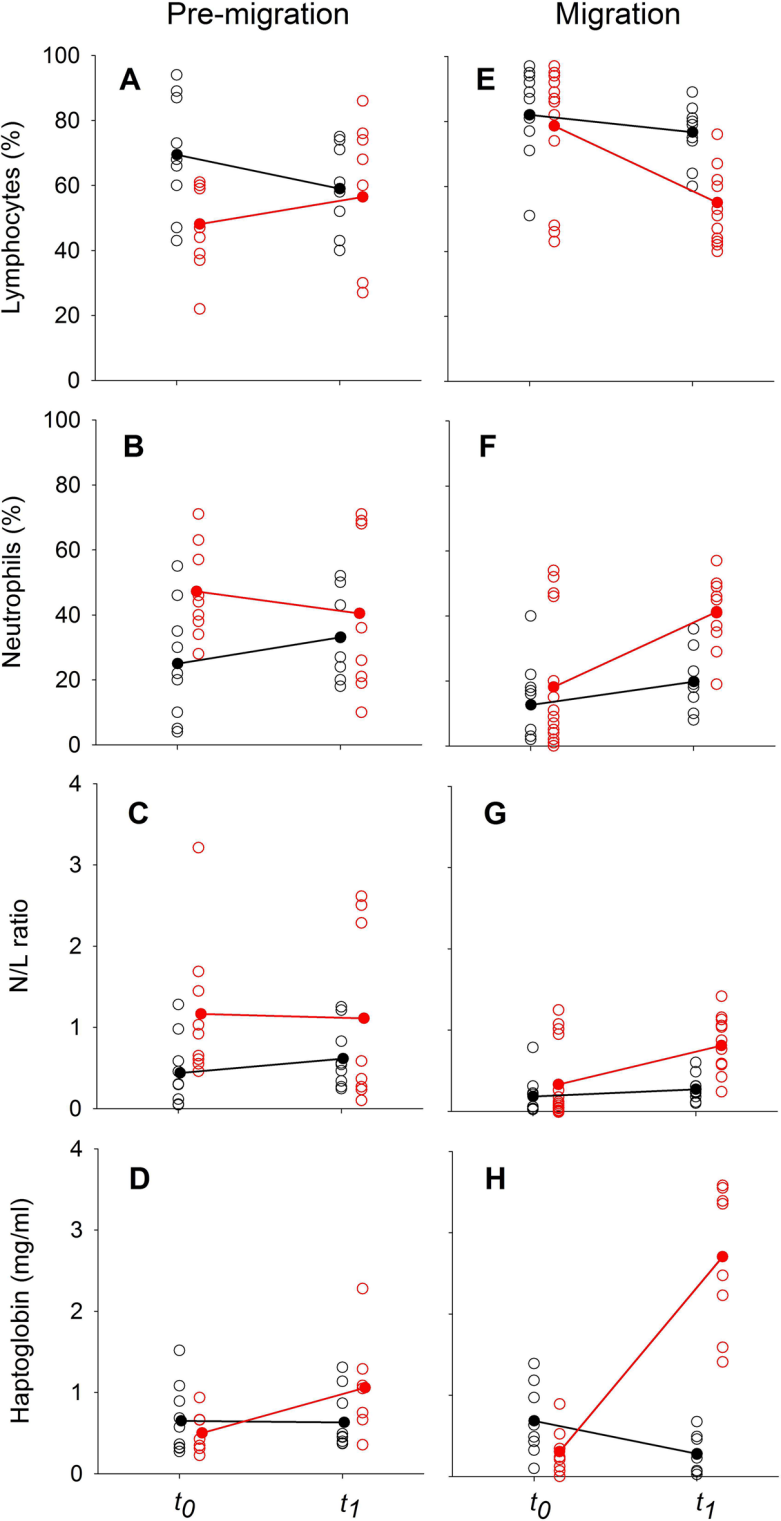
Changes in the relative number of white blood cells (%;lymphocytes; **A, E**; neutrophils; **B, F**), N/L ratios **(C, G)** and haptoglobin concentration (mg ml^-1^; **D, H**) in bats of the experimental (LPS, red) and control group (black) during the pre-migration (left graphs) and migration season (right graphs).

**Figure 4:**
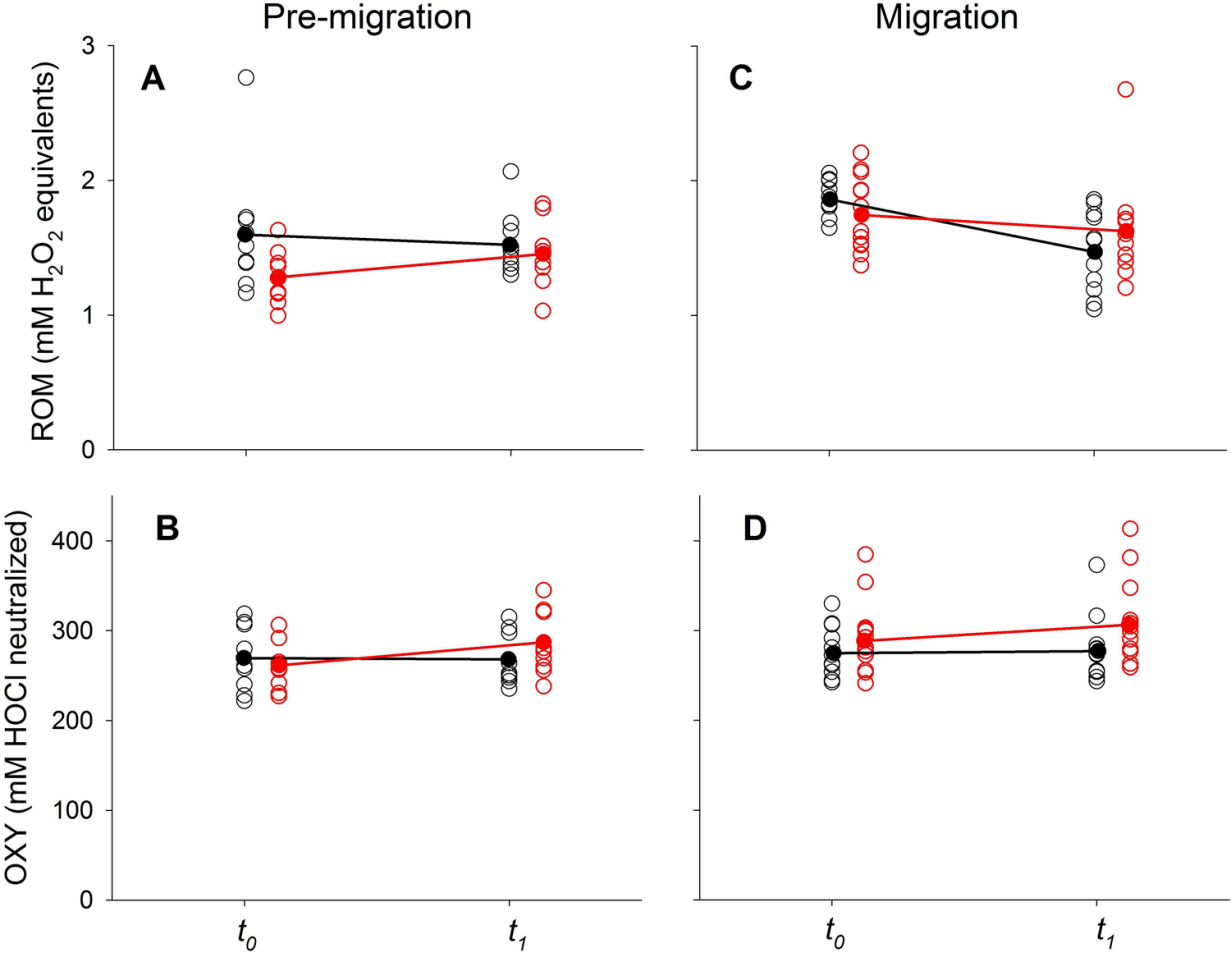
Changes in primary plasma oxidative damage products measured as ROMs (mM H_2_0_2_ equivalents; **A, C**) and plasma non-enzymatic antioxidant capacity measured as OXY (mM HOCl neutralized, **B, D**) in bats of the experimental (LPS, red) and control group (black) during the pre-migration (left graphs) and migration season (right graphs).

During the *pre-migration period*, the proportion of lymphocytes in blood smears of *P. nathusii* differed between treatments (SE = 0.66, z = 2.5, p = 0.014) prior to the injection (all raw data in supplementary material). Following the LPS challenge, the proportion of lymphocytes increased significantly in relation to other immune cells in experimental animals but not in control bats (SE = 0.41, z = − 2.0, p = 0.046, Fig. 3A, Table 2). The proportion of neutrophils differed between groups (SE = 0.71, z = − 2.5, p = 0.031), but changes over time remained not significant between groups (SE = 0.45, z = 1.6, p = 0.109, Fig. 3B). LPS challenged bats had higher N/L ratios than control animals (SE = 0.68, z = − 2.04, p = 0.041), but we did not observe a difference in the change of N/L ratios between groups during the experiment (SE = 0.42, z = 1.48, p = 0.140, Fig. 3C). Plasma haptoglobin concentrations did not differ between groups (sqrt, SE = 0.26, t = 1.47, p = 0.155) and we also did not observe a difference in the change of plasma haptoglobin concentrations between groups during the experiment (SE = 0.15, t = − 1.57, p = 0.142; Fig. 3D). We could not find differences in ROM levels between groups prior to the immune challenge (SE = 0.26, t = − 0.9, p = 0.406) and no difference in changes between groups during the experiment (SE = 0.16, t = 0.3, p = 0.735, Fig. 4A). Similarly, we did not observe differences in the OXY levels between treatments (SE = 26.6, t = − 1.1, p = 0.301) and no difference in changes between groups during the experiment (SE = 15.1, t = 1.56, p = 0.143; Fig. 4B). Body masses decreased over time in both groups (SE = 0.107, t = − 4.87, p < 0.001), yet body mass changes did not differ between groups (SE = 0.15, t = − 0.73, p = 0.474).

**Table 2:** Change in immunological parameters and measures of oxidative status in LPS challenged and control animals of *P. nathusii* from Latvia during the pre-migration and migration season. Values indicate mean ± one standard deviation (median).

	Pre-migration		Migration	
	Control	LPS	Control	LPS
Lymphocytes (%)	− 10.4 ± 15.3 (− 14.0) n = 9 pairs	8.3 ± 23.7 (10.0) n = 8 pairs	− 4.2 ± 14.1 (− 7.0) n = 11 pairs	− 25.4 ± 19.2 (− 27.0) n = 13 pairs
Neutrophils (%)	8.1 ± 15.5 (13) n = 9 pairs	− 7.1 ± 25.9 (− 11.0) n = 8 pairs	6.2 ± 13.6 (12.0) n = 11 pairs	25.1 ± 18.4 (32.0) n = 13 pairs
N/L ratio	0.18 ± 0.45 (0.23) n = 9 pairs	− 0.09 ± 1.14 (− 0.37) n = 8 pairs	0.07 ± 0.25 (0.17) n = 11 pairs	0.52 ± 0.47 (0.54) n = 13 pairs
Haptoglobin (mg/ml)	− 0.02 ± 0.58 (0.08) n = 9 pairs	0.56 ± 0.46 (0.44) n = 7 pairs	− 0.41 ± 0.49 (− 0.33) n = 8 pairs	2.40 ± 1.04 (2.58) n = 13 pairs
ROMs (mM H_2_0_2_ equivalents)	− 0.08 ± 0.49 (− 0.10) n = 9 pairs	0.19 ± 0.29 (0.33) n = 8 pairs	− 0.36 ± 0.36 (− 0.25) n = 10 pairs	− 0.05 ± 0.40 (− 0.16) n = 8 pairs
OXY (mM HOCl neutralized)	− 1.5 ± 14.3 (− 3.5) n = 9 pairs	25.6 ± 43.6 (13.2) n = 9 pairs	− 2.6 ± 20.5 (− 7.8) n = 9 pairs	22.4 ± 36.7 (13.3) n = 11 pairs

During the *migration period*, we did not observe differences between treatment groups for the proportion of lymphocytes (SE = 0.62, z = 0.3, p = 0.75) or temporal changes in the LPS treated group compared to the control group (SE = 0.38, t = − 1.6, p = 0.114; Fig. 3E; Table 2) (all raw data in supplementary material). The proportion of neutrophils did not differ between treatment groups (SE = 0.68, z = 0.7, p = 0.5), and we did not observe differences in changes of neutrophil proportions during the experiment between groups (SE = 0.41, z = 0.7, p = 0.486; Fig. 3F). We did not observe a difference in the N/L ratio between bats of the LPS and control group (SE = 0.67, z = 0.51, p = 0.613). Also, we could not find a difference in the changes over time between groups (SE = 0.39, z = 0.82, p = 0.411; Fig. 3G). Plasma haptoglobin concentrations differed between treatment groups (Tukey’s ladder of powers transformed, SE = 0.16, t = − 4.3, p < 0.001). Plasma haptoglobin concentrations increased by a factor of 7.8 in relation to baseline values in migratory bats, whereas they remained similar throughout the experiment in bats during the pre-migration period (increase 2.4 ± 1.04; SE = 0.1, t = 4.9, p < 0.001; Fig. 3H). We did not observe differences in the overall ROM levels between treatments (SE = 0.252, t = − 1.7, p = 0.106), but significant decreases of ROM levels over time in both groups (SE = 0.161, t = − 2.2, p = 0.035, Fig. 4C). Lastly, we did not observe differences in OXY levels between treatments (SE = 26.166, t = − 1.1, p = 0.266) and no difference in the increase of OXY levels between groups (SE = 13.706, t = 1.8, p = 0.082, Fig. 4D). We found differences in body masses between groups prior to the experiment (SE = 0.26, t = 2.7, p = 0.009) and a decrease of body mass over time for both groups (SE = 0.08, t = − 13.7, p < 0.001). Body mass decreases differed between the experimental and control groups (SE = 0.11, t = − 2.19, p = 0.039).

## Discussion

Our study shows that Nathusius’ pipistrelle bats adjust their immune system when switching from the pre-migratory to the migratory season. Contrary to our prediction, baseline proportions of lymphocytes (part of the adaptive immunity) were higher and those of neutrophils (cellular effectors of the innate immune system) lower towards the migration season compared to the pre-migration season. A lower N/L ratio in migratory Nathusius’ pipistrelles than in pre-migratory conspecifics suggests a lower inflammatory state in bats during the migration season. Plasma haptoglobin concentrations were similar in bats of the pre-migration and migration season. Higher proportions of lymphocytes in relation to other immune cells in migratory bats might be indicative of a higher relevance of the adaptive cellular immunity for migratory Nathusius’ pipistrelles, yet based on the use of cell counts we cannot infer on the absolute concentration of lymphocytes in the blood of migratory bats. The proportion of lymphocytes did not change in LPS treated bats during the migration period; yet lymphocyte proportions increased in bats during the pre-migration period. This observation questions a functional role of lymphocytes during an acute phase response in migratory bats. Plasma haptoglobin levels increased by a factor of 7.8 in relation to baseline values in LPS challenged bats during the migration. During the pre-migration season, plasma haptoglobin levels did not change significantly in LPS challenged bats. This result suggests that Nathusius’ pipistrelles, and possibly also other migratory bats, are prudent with energetically costly cellular immune responses during migration in order to save energy. Additionally, they may boost both their baseline and functional immunity during migration in order to decrease costs of mounting an immune response during this period (e.g. to produce immune cells).

We acknowledge that our experimental design may have suffered from a low sample volume and size which hindered us in measuring leucocyte concentrations and which may have masked small effects of the treatment, respectively. Yet, considering that previous challenge experiments in bats revealed significant responses of the cellular immunity with similar sample sizes^40,41^, we find it robust to conclude that in migrating Nathusius’ pipistrelles an acute phase response is largely driven by humoral but not cellular immunity. Additionally, we recognize potential limitations of our study that may arise from including only females during the pre-migration and both sexes during the migration season. This was caused by the fact that we did not encounter any male at our study site during the pre-migration period. Yet since females were in a post-reproductive state, we do not anticipate large differences in the immune response of females compared with non-reproductive males; particularly since similar studies did not observe an effect of sex on the acute phase response^47–50^. Therefore, we consider it unlikely that a sex-bias in our sampling design confounded our results.

Haptoglobins are faster and cheaper to produce compared to cellular effectors, thus haptoglobin may be selected as the first line of defence when bats are challenged during extreme physiological conditions. Similar results have been described for hibernating bats when challenged with zymosan, a fungal antigen^47^. Alternatively, we may have missed the response of cellular immunity by covering only a 24 h period post-injection. However, previous studies using a similar experimental design showed that the cellular immunity of bats responded to an LPS challenge within 24 h^38^ or even shorter periods (8 h)^49^. Since we did not cover more than 24 h in our experiment, we cannot rule out that plasma haptoglobin values may even reach higher values after more time has elapsed. Possibly, the activation of the cellular innate immunity might involve more metabolic costs and may thus force bats to rest for a prolonged period for a comprehensive cellular immune response. An extended stopover of challenged migratory animals has been confirmed for birds^50^. Immunological data collected in our study from Nathusius’ pipistrelles is most consistent with the idea of an energetic trade-off in migratory bats favouring the humoral innate immune branch over the cellular branch in a challenge situation.

We confirmed a higher oxidative damage in migrating than in pre-migrating Nathusius’ pipistrelles^11^, but observed no effect of the LPS treatment on plasma oxidative status markers in both migrating and pre-migrating Nathusius’ pipistrelles. Moreover, the level of damage was not correlated with the main immune cell counts. These results confirm that the intense physical exercise of migratory flight results in high levels of oxidative stress^11^, most likely caused by the production of reactive oxygen species in mitochondria. Inflammation, which may be caused by intense physical effort and which may contribute to oxidative stress, does not appear to be relevant here, because haptoglobin concentration was similar between migrating and pre-migrating bats and because N/L ratios were low in migratory bats. Haptoglobin is considered to be an inflammatory protein which responds tightly to inflammatory processes and is effective against microbial pathogens. The lack of a potential constraining effect of oxidative stress on immunity might be caused by the transient condition in which bats stayed. Previously, we observed that, while resting, oxidative damage diminishes rapidly^11^. Thus, resting bats seem to be able to rapidly detoxify their body from accumulation of oxidized biomolecules.

Our results are partly consistent, partly contrasting with previous findings from migratory birds. For example, Eikenaar and Hegemann showed that baseline values for the innate humoral branch were lower in migratory than in resident blackbirds (*Turdus merula*)^51^. Parameters of the adaptive humoral immunity were similar in both groups^51^. Unfortunately, the authors did not challenge study birds with an antigen so that it remains unclear if these branches would remain low during an acute phase response. Owen and Moore showed that the cellular immune response of Swainson’s thrushes to an immune challenge was suppressed in migratory compared to pre-migratory individuals, suggesting a trade-off situation in which cellular immunity was down-regulated in favour of migration^52^, similar to the results of our study.

## Conclusion

Based on our study, we infer that migrating Nathusius’ pipistrelles may not suppress all branches of their immunity during the costly activity of migration. Instead, they seem to selectively downregulate the functional role of cellular effectors, which might help to save energy for their annual journeys. Thus, Nathusius’ pipistrelles seem to depend more strongly on the response of the humoral immunity when encountering pathogens during migration.

## Material and methods

### Field sites

Field work was carried out in Latvia under the licenses 10/2015 and 31/2016-E, issued by the Latvian Nature Conservation agency. All methods were carried out in accordance with general guidelines^53^. Experimental protocols were approved by the animal care and welfare committee of the Leibniz Institute for Zoo and Wildlife Research. During the pre-migration season, we performed experiments from 11th to 13th of July 2016 at Engure Ornithological Station (EOS; 57°15′34′′ N 23°08′08′′ E, Engure municipality). Since we did not encounter male *P nathusii*, we used exclusively adult females for this experiment. All females were in a post-reproductive condition. At that time, juveniles were already weaned and foraged independently of their mothers. Between 10 and 11 pm, we transferred 18 female *P. nathusii* from wooden bat boxes installed near EOS to holding boxes. During the migration season, we captured 28 adult *P. nathusii* (11 females, 17 males) in mid-August (16 and 18 August 2015) between 10 pm and 1 am in the funnel trap located next to Pape Bird Ringing Station (PBRS; 56°09′57′′ N 21°01′02′′ E, Rucava municipality). All females were in a post-lactating condition and males in a pre-reproductive condition, with small testes and epididymis. EOS and PBRS are separated by about 180 km. All bats captured at PBRS defecated after capture and therefore we assumed that bats had recently fed on insects. Bats at EOS were captured prior to foraging and therefore we fed all bats with mealworms and offered water prior to experiments. Consequently, bats entered the experiment at both sites in a fed condition. Further, experiments at EOS and PBRS were conducted at the same time of the day to control for diurnal effects on immunity^43^.

### Experimental procedure

After capture, we immediately brought bats to the nearby field station where we determined the sex and measured body mass (0.1 g accuracy, digital balance) and forearm length (0.1 accuracy, manual calliper). For individual identification, we marked bats using special bat bands. We processed bats one by one during the night of capture. First, we took an initial (pre-treatment) blood sample of about 60 µl by puncturing the uropatagial vein of bats using a sterile disposable needle (40 gauge) and heparinized capillary tubes. We used a droplet of blood to produce a blood smear on a glass slide (microscope slides; 76 × 26 mm, cut edges, Menzel, 38116 Braunschweig Germany). The rest of the blood sample was centrifuged and plasma separated from the blood cells using heparinized capillary tubes. Both the blood cells and plasma samples were kept cold on ice packs for about 5–10 min until they were transferred to a dry shipper in which they stayed until they reached the laboratory in Berlin, Germany, where they were stored at − 80 °C until further analysis.

Bats were divided randomly into an LPS and a control group. In bats of the LPS group, we injected subcutaneously with sterile disposable syringes 25 µl of 1 mg ml^−1^ LPS from *Escherichia coli* O111:B4 (no. L2630, Sigma-Aldrich, Munich, Germany) solved in isotonic NaCl solution (Carl Roth GmbH, Karlsruhe, Germany). LPS is an endotoxin that induces an inflammatory immune reaction in treated animals, as well as sickness behaviour such as reduced locomotion and feeding behaviour^54^. Used antigen dosages were adjusted to the species’ body mass and similar to those reported before in experiments with bats^38^. We injected a 25 µl isotonic saline solution in each individual of the control group. Afterwards, all bats were transferred into a keeping box where they rested for 24 h in a separate compartment. Here, bats were exposed to ambient temperature (10–20 °C) and humidity (60–80%) in complete darkness. In order to ensure that bats maintained a similar body mass at the time of bleeding, we fed all bats after about 22 h post-injection with mealworms and offered water from a syringe. At 24 h post-injection, we measured the body mass of bats and then took a second blood sample of about 50 µl from the uropatagial vein. Similar to the initial bleeding event, we produced blood smears, separated plasma from the blood cells, and stored the samples in similar conditions. Afterwards, all bats were released at their respective field site following another opportunity to feed or drink and a general health check.

### White blood cell counts

We stained blood smears with May-Gruenwald’s solution (no. T863.2, Carl Roth GmbH, Karlsruhe, Germany) and Giemsa (no. T862.1, Carl Roth GmbH, Karlsruhe, Germany). We conducted differential white blood cell (DWBC) counts by counting 100 leukocytes under 1000 × magnification (oil immersion) and by calculating the relative numbers of lymphocytes and neutrophils; we did not consider monocytes, basophils and eosinophils due to low concentrations^55^. We attempted to estimate the total number of white blood cells by counting the mean number of immune cells in 10 visual fields with a microscope under 200 × magnifications^55^. However, the number of WBC was close to zero, therefore we only used the DWBC in the statistical analysis.

### Haptoglobin analysis

Haptoglobin is an acute phase protein that usually occurs at low concentrations, but production and secretion is increased in response to acute infection and trauma^56^, including in bats^47,57^. As an acute phase protein, haptoglobin reduces oxidative damage by binding hemoglobin released during hemolysis, has immunomodulatory effects and inhibits bacterial growth. To measure the concentration of plasma haptoglobin, we followed the standard procedure of the commercial kit "PHASE" Haptoglobin Assay (Cat. No. TP-801, Tridelta, Maynooth, Ireland) using a colorimetric assay previously described for other bat species^39,47^.

### Measures of blood oxidative status

We measured one marker of plasma oxidative damage and one marker of plasma non-enzymatic antioxidant capacity using standard methods for vertebrates recently applied to *P. nathusii*^11^ and other bat species^39,48^. Prior work showed that both markers may be significantly affected by stimulation of immune function^38^ or by the physical activity^58^. Briefly, primary oxidative damage products (e.g., organic hydroperoxides, endoperoxides) were measured in plasma using the d-ROMs assay (Diacron International, Grosseto, Italy). Values were expressed as mM H_2_O_2_ equivalents. The non-enzymatic antioxidant capacity was measured using the OXY-Adsorbent test (Diacron International). This assay quantifies the in vitro reaction between the non-enzymatic antioxidants (e.g., vitamins, thiols, carotenoids) that occur in plasma (diluted 1:100 with distilled water) and the oxidant hypochlorous acid (HOCl, an endogenously oxidant produced by mammalian neutrophils). Values were expressed as mM of HOCl neutralised. All analyses were carried out in duplicate.

### Statistical analysis

We performed statistical analyses either with SYSTAT (vs. 11, SYSTAT Software GmbH; Erkrath, Germany) or the open source software R (R core group 2019, version 3.6.2., R Development Core team, Vienna Austria). Owing to limitations in volume and cell numbers, we only compared differential leukocyte counts and plasma haptoglobin between the first and second bleeding event. Two outliers were detected by using Cook's distance in dROM data and removed from the analysis (one in pre-migration and one in migration season). To test for an effect of LPS treatment during pre-migration and migration season, we used beta regressions (betareg package)^59^ for relative numbers of lymphocytes and neutrophils (response ~ treatment group * day of treatment + body mass * FA) and linear mixed models fitted by REML (lmerTest package)^60^ for concentrations of haptoglobin, ROMs, and OXY (response ~ treatment group * day of treatment + body mass * FA + (1 | individual = random effect). All response variables,) were tested for normal distribution of the residuals (Shapiro–Wilk test). Non-normal distributed data (e.g. haptoglobin data) were transformed per square root (sqrt) or Tukey’s ladder of Powers into normal distributions and models were analyzed using Satterthwaite's t-test. In case of non-normal distributed data or residuals in the models we used Wilcoxon–Mann–Whitney-tests, e.g. for comparisons of blood parameters between both seasons. Correlations were calculated using the Spearman correlation test. We set significance levels to alpha = 0.05 for all analysis. Parameters are presented as means ± one standard deviation if not stated otherwise. All figures were made with SigmaPlot (Version 13.0, Systat Software, San Jose, CA).

## Supplementary information

Supplementary Information.

## Data Availability

All data generated or analysed during this study are included in this published article (and its Supplementary Information files).
